# Field assessment of a novel spatial repellent for malaria control: a feasibility and acceptability study in Mondulkiri, Cambodia

**DOI:** 10.1186/s12936-017-2059-6

**Published:** 2017-10-13

**Authors:** Marco Liverani, Jacques Derek Charlwood, Harriet Lawford, Shunmay Yeung

**Affiliations:** 1 Department of Global Health and Development, London School of Hygiene and Tropical Medicine, London, UK; 2 Liverpool School of Tropical Medicine, Liverpool, UK; 3 Department of Clinical Research, London School of Hygiene and Tropical Medicine, London, UK

**Keywords:** Malaria, Cambodia, Spatial repellent, Metofluthrin, Residual malaria transmission, Disease awareness, Malaria protective measures, Social and economic aspects of malaria control

## Abstract

**Background:**

Large-scale use of insecticide-treated nets and indoor residual spraying have contributed to a significant decrease in malaria transmission worldwide. Further reduction and progress towards elimination, however, require complementary control measures which can address the remaining gaps in protection from mosquito bites. Following the development of novel pyrethroids with high knockdown effects on malaria vectors, programmatic use of spatial repellents has been suggested as one potential strategy to fill the gaps. This report explores social and contextual factors that may influence the relevance, uptake and sustainable use of a spatial repellent in two remote villages in Mondulkiri province, Cambodia, with endemic malaria transmission. The repellent consisted of polyethylene emanators, held in an open plastic frame and impregnated with 10% metofluthrin.

**Results:**

In a baseline survey, 90.9% of households in Ou Chra (n = 30/33) and 96.6% in Pu Cha (n = 57/59) were interviewed. Behavioural data were collected for all household occupants (n = 448). In both villages, there were times and places in which people remained exposed to mosquito bites. Prior to the installation of the repellent, 50.6 and 59.5% of respondents noted that bites occurred “very often” inside the house and in the outdoor area surrounding the house, respectively. Indoor biting was reported to occur more frequently in the evening, followed by at night, while outdoor biting occurred more frequently in the early morning. In a follow-up survey, spatial repellents were well received in both villages, although 63.2% of respondents would not replace bed nets with repellents. Most participants (96.6%) were willing to use the product again; the mean willingness to pay was US$ 0.3 per unit. A preference for local procurement methods emerged.

**Conclusion:**

Widespread use of spatial repellents would not fill all protective gaps, but, if their entomological efficacy can be ascertained, outdoor application has the potential to enhance vector control strategies in Cambodia. Successful implementation would require subsidisation and integration with the existing national malaria control strategy. It is hoped that this study, while contributing to a better understanding of the social contexts of residual malaria transmission, will generate further interest in the evaluation of spatial repellents for malaria control.

**Electronic supplementary material:**

The online version of this article (doi:10.1186/s12936-017-2059-6) contains supplementary material, which is available to authorized users.

## Background

Large-scale distribution of insecticide-treated nets (ITNs) and indoor residual spraying (IRS) have been prioritized as the main methods for the control of malaria vectors worldwide [1]. Evidence indicates these approaches have contributed to a substantial decline in malaria transmission [2]. However, there is wide recognition that further reduction and progress towards malaria elimination require innovative and complementary approaches [3]. Even with optimal ITN coverage, residual malaria transmission may continue as vectors feed before sleeping times, both outdoors and indoors, when ITNs are not in use and people are not protected [4]. In addition, some vector species may enter houses to feed and then rest outdoors, avoiding fatal exposure to insecticide treated surfaces [5]. Interventions such as ITNs and IRS may also be unsustainable in the long term due to their dependence on certain pyrethroid classes, to which many mosquitoes have developed resistance [6]. In light of this, it is apparent that alternative tools that can address protective gaps are needed [7].

Programmatic use of spatial repellents has been suggested as one potential strategy to fill these gaps [8], especially after the recent development of novel active ingredients. Metofluthrin, for example, is a new volatile pyrethroid with high ‘knockdown’ effects on malaria vectors and relative longevity, which make it suited for use as a spatial repellent [9]. Several arguments can support interventions based on this method. First, novel spatial repellents may be effective in both indoor and outdoor environments [10, 11], thus would be useful to ensure continued protection when ITNs and IRS are not applicable. Second, unlike topical repellents which must be applied daily, consistent and appropriate use of spatial repellents does not require high levels of user compliance. Third, research has demonstrated the effectiveness of spatial repellents against multiple vectors and species of vectors, and consequently they may help reduce other diseases, such as dengue fever [11].

However, the success of vector control programmes is not solely dependent on the entomological effects of proposed interventions, but requires uptake and use by the target population. Whether such products will be adopted and useful requires an understanding of social and contextual factors that may influence product acceptability and adequacy [12]. These include risk perceptions, disease awareness, previous exposure to other control measures, the spatial and temporal dimension of household routines and other social practices. In addition, user preference for cost and delivery methods may have considerable impact on product uptake and sustainability.

In the present report, we explore these issues by examining the feasibility and acceptability of spatial repellents in two rural communities in Mondulkiri province, Cambodia. Following a nationwide scale up in malaria control interventions, including prevention methods and case management, the total number of reported malaria cases in this country has declined significantly from 129,167 in 2000 to 23,627 cases in 2016 [13]. In the wake of this success, the Ministry of Health in Cambodia has called for the elimination of *Plasmodium falciparum* malaria by 2020 and a phased elimination of all forms of malaria by 2025 [14]. Further, sustained efforts have been made to contain and eliminate artemisinin-resistant malaria, which was first reported in 2006 in Western Cambodia along the border with Thailand [15, 16]. However, the proportion of malaria infections occurring before sleeping hours or outdoors remains an important challenge to elimination [4, 17]. Controlling outdoor transmission of malaria is of particular importance in Cambodia, where the most prominent vectors are exophilic and where the population spends a high proportion of time outdoors, especially during peak biting times [18]. This challenge is most critical amongst ethnic minorities, migrants and mobile populations in remote locations near forested areas, who face the highest risk of malaria infection. Thus, it is important to develop interventions and vector control measures that are acceptable, feasible, and sustainable to use amongst these population groups.

In this context, a social science study was conducted to understand the reception and feasibility of a metofluthrin spatial repellent in two remote communities, where endemic malaria transmission occurs. This study was developed and implemented in coordination with an entomological and parasitological survey in the same villages, which aimed to assess the efficacy of this product. Here, the findings from the social science study are presented and discussed.

## Methods

### Study site and population

The study was conducted in two small villages in Mondulkiri province, Ou Chra and Pu Cha, with a population of 150 and 312, respectively. Ou Chra (12.235721, 106.848192) and Pu Cha (12.207171, 106.857025) are located in forested areas near the eastern border with Vietnam along a stretch of dirt road, circa 15 and 20 km respectively from Kaev Seima town. These villages were chosen as they represent a typical scenario of endemic malaria transmission in Cambodia—remote communities of ethnic minorities, living in proximity to tropical forests where the malaria vectors breed [19].

The great majority of residents in both villages are ethnic Phnong (also known as Bunong), an aboriginal group mainly found in Mondulkiri that rely on subsistence agriculture [20]. Malaria transmission in the province occurs throughout the year at relatively low rates, with peaks during the rainy season (June–July to October–November). The natural forest cover in a radius of 2 km around Ou Chra reduced from 98.0% in 2010 to 77.4% in 2015 [21]. The only road in and out the villages becomes nearly impassable during the rainy season, isolating the communities from the nearest market town and public health facilities. Both Ou Chra and Pu Cha have village malaria workers (VMWs), community health volunteers who are trained to diagnose suspected malaria cases by using a rapid diagnostic test (RDT), administer artemisinin-based combination therapy, and refer patients to the nearest public health facility [22, 23]. Long lasting insecticide-treated bed nets (LLINs) are distributed free of charge by the national malaria programme, and, as in the rest of Cambodia, their continued availability represents the main vector control method [14, 24].

### Study design and data collection

In 2013, a cross-over entomological and parasitological survey was conducted in both villages to assess the effects of the spatial repellent metofluthrin (2,3,5,6-tetrafluoro-4-(methoxymethyl)benzyl(*EZ*)-(1*RS*)-cis–trans-2,2-dimethyl-3-prop-1-enylcyclopropanecarboxylate), supplied by Sumitomo Chemical Co. Ltd. (Hyogo, Japan), on anopheline densities and malaria incidence and prevalence. At the start of the study all houses in Ou Chra (n = 33) and Pu Cha (n = 59) were mapped with hand held GPS units. The emanators were made of polyethylene dual layer (15 × 8 cm wide) mesh held in an open plastic frame impregnated with 10% (w/w) metofluthrin. According to the manufacturers, a single emanator can protect a 30 m^3^ space for 4 weeks. The total estimated volume of each area requiring protection was determined, the number of emanators required and their optimum location (such as by the eave gap, if there was one) established. They were generally installed above head height so that they did not interfere with other activities and were themselves out of reach of curious children. The mean allocation of repellents per household was 2.5, with a range between 1 and 6 units depending on the household and room size.

In coordination with the entomological and parasitological survey, whose findings are presented in a previously published paper [25], an exploratory social science study was conducted in both villages to assess the feasibility and acceptability of the spatial repellent in the communities. The research design involved two stages of data collection, aiming to cover all households. In June 2013, prior to the first instalment of the emanator, a baseline survey was undertaken in both villages to gain an understanding of risk factors and protective gaps as well as social and contextual variables that may influence product uptake and utilization. The baseline questionnaire focused on risk perceptions and disease awareness and the use of other protective measures against mosquito bites, including details on ITN utilization, procurement, and cost (Additional file 1). Respondents were then asked to indicate the location of all household members at different periods of the day preceding the interview, according to local understanding of time (Additional file 2): *bpro leum* (early morning, 5:00 to 7:00), *broek* (morning, 7:00 to 12:00), *rosial* (afternoon, 12:00 to 17:00), *l’ngiak* (evening, 17:00 to 21:00), and *yub* (night, after 21:00). Information on socio-economic indicators was also collected to produce an index of socio-economic status, which was used subsequently to test potential differences in willingness to pay (WTP) for the emanator across population groups. Socio-economic variables included ownership of assets (i.e. radio, television, mobile phone, cupboard, bicycle, cart, tractor, and motorcycle), agricultural land, electricity, source of water, and house construction materials. Notes were taken during field trips to record observations about housing characteristics, household assets, and informal conversations with the villagers about social practices in the communities. One month after the first instalment of the spatial repellent, a follow-up survey was conducted in the same households to assess reception and acceptability of the product (in August in Ou Chra and in November in Pu Cha). In keeping with past evaluations of malaria control measures [26, 27], the second survey focused on perceptions about the effectiveness of the product, WTP and preferences for different procurement mechanisms (Additional file 3). Open-ended questions were included in both questionnaires to elicit further input and explanations on structured questions. Estimates of individual WTP were elicited with an iterative ‘bidding game’ approach [28].

Questionnaires were administered to the female head of household or another household member (where the female head was not available). Female heads of households were prioritized as women in rural Cambodia are usually responsible for day-to-day household management, small household purchases, and the management of bed nets [29, 30]. Most interviews took place in the morning, but some took place in the afternoon or early evening, depending on the participants’ availability and whether they were at home/in the village at the time of interview. Participants were interviewed face-to-face at their homes, and were either alone or in the presence of their spouse or other relative. The two surveys were carried out by a team of four researchers, all graduates in the social sciences and native Cambodian speakers, in close coordination with ML, who participated in the early field trips.

### Data management and analysis

Survey data were double-entered and cleaned using an EpiData template. The datasets were then imported into STATA version 13 (StataCorp, College Station, TX, USA) for descriptive analysis and test statistics. Textual information from open-ended responses was translated into English, entered into an Excel file, linked to the survey dataset by unique identifiers, and coded for frequency analysis. A response was given multiple codes if more than one explanation was provided. Principal component analysis (PCA) was used to generate a relative wealth index, based on household-level information on socio-economic indicators [31, 32]. The first principal component was used to derive weights for wealth index. Households were then divided into tertiles based on their individual score against the wealth index: poorest, poor, and least poor. The Kruskal–Wallis test was used to test for significant differences in mean WTP across socioeconomic groups; an independent t test was used to examine associations between gender and WTP. Field notes were discussed collectively at the end of field trips, and reports were written. Key points from field reports are integrated into the presentation of results below to complement findings from the survey analysis.

## Results

### Demographic, socio-economic, and housing characteristics

Thirty of 33 households (90.9%) in Ou Chra and 57 of 59 (96.6%) in Pu Cha were interviewed. Characteristics of the study population (n = 448) are summarized in Table 1. Occupation, educational level, and socio-economic status did not vary significantly between the two villages. The great majority of residents with an occupation were farmers (92.4%), with an almost equal proportion of men (50.9%) and women (49.1%) involved in agricultural work. The mean length of residence of adults in Ou Chra was 23.9 years while in Pu Cha it was 14.1 years.

**Table 1:** Demographic and socioeconomic characteristics of all occupants in surveyed households

	Ou Chra n (%)	Pu Char n (%)	Totals n (%)	p value
Age group (years)^a^				0.241
< 5	12 (8.9)	48 (15.3)	60 (13.4)	
5–12	37 (27.4)	73 (23.3)	110 (24.6)	
13–35	62 (45.9)	130 (41.5)	192 (42.9)	
> 35	24 (17.8)	62 (19.8)	86 (19.2)	
Gender^a^				0.952
Female	66 (48.9)	154 (49.2)	220 (49.1)	
Male	69 (51.1)	159 (50.8)	228 (50.9)	
Education^a^				
(Some) primary	67 (49.6)	149 (47.6)	216 (48.2)	0.706
(Some) secondary or other	18 (13.3)	36 (11.5)	54 (12.05)	
No education	50 (37.0)	128 (40.9)	178 (39.7)	
Occupation^a^				0.468
Farmer	65 (48.2)	153 (48.9)	218 (48.7)	
Student or child	58 (43.0)	142 (45.4)	200 (44.64)	
Other or none	12 (8.9)	18 (5.6)	30 (6.7)	
Residency status^b^				1.000
Resident	134 (100)	311 (99.4)	445 (99.6)	
Visitor	0	2	2 (0.5)	
Own at least one^a^				
TV	13 (43.3)	20 (35.1)	33 (37.9)	0.451
Radio	7 (23.3)	8 (14.0)	15 (17.2)	0.275
Mobile phone	15 (50)	40 (70.18)	55 (63.22)	0.064
Bicycle	5 (16.7)	4 (7.0)	9 (10.3)	0.160
Moto	26 (86.7)	73.7	68 (78.2)	0.164
Tractor	14 (46.7)	17 (29.8)	31 (35.6)	0.119
HH socio-economic status^a^				0.137
1 (poorest)	9 (30.0)	20 (35.1)	29 (33.3)	
2 (poor)	7 (23.3)	22 (38.6)	29 (33.3)	
3 (least poor)	14 (46.7)	15 (26.3)	29 (33.3)	

^a^Chi square

^b^Fisher’s exact test

**Fig. 1:**
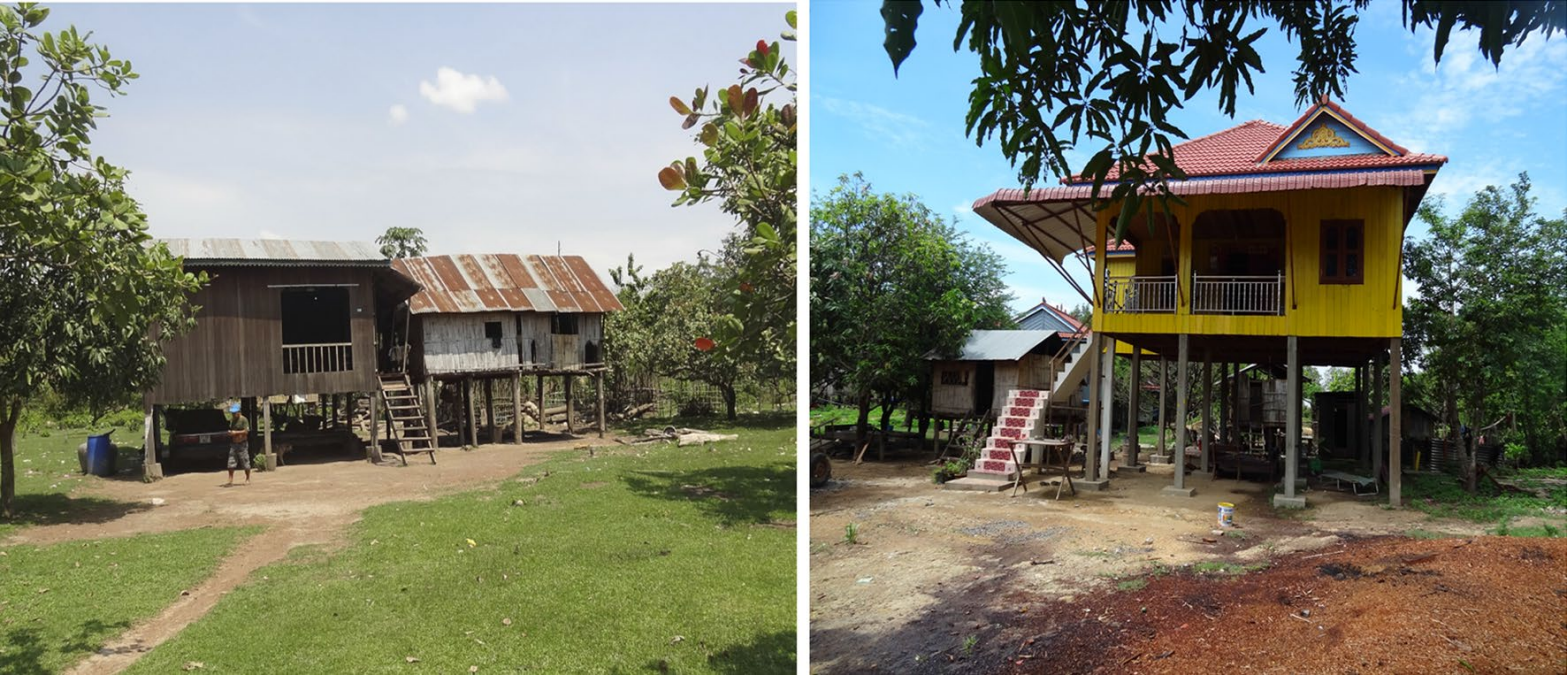
Examples of different types of houses in Ou Chra (Photos: JD Charlwood)

As observed in both villages, houses were built in a typical Khmer style, but differed in their quality of construction (Fig. 1). Most houses were relatively well built on stilts, but some were built on the ground or in a bad state of repair with large gaps in the walls. The exterior of the houses was reflected in the interior. People living in well-built houses had many more material possessions such as motor vehicles, televisions or radio, while the inhabitants of houses in disrepair had very little. In both villages, televisions (or DVD players) were common. Some farmers also had a second house or shelter in their rice fields or farms, where they could stay overnight during the harvesting season. These dwellings were partly or completely open, providing easy entry to mosquitoes [33].

### Household routines

**Fig. 2:**
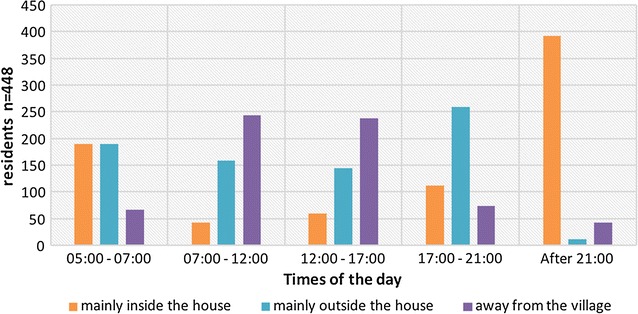
Location of residents in Ou Chra and Pu Cha at different times of the day

**Fig. 3:**
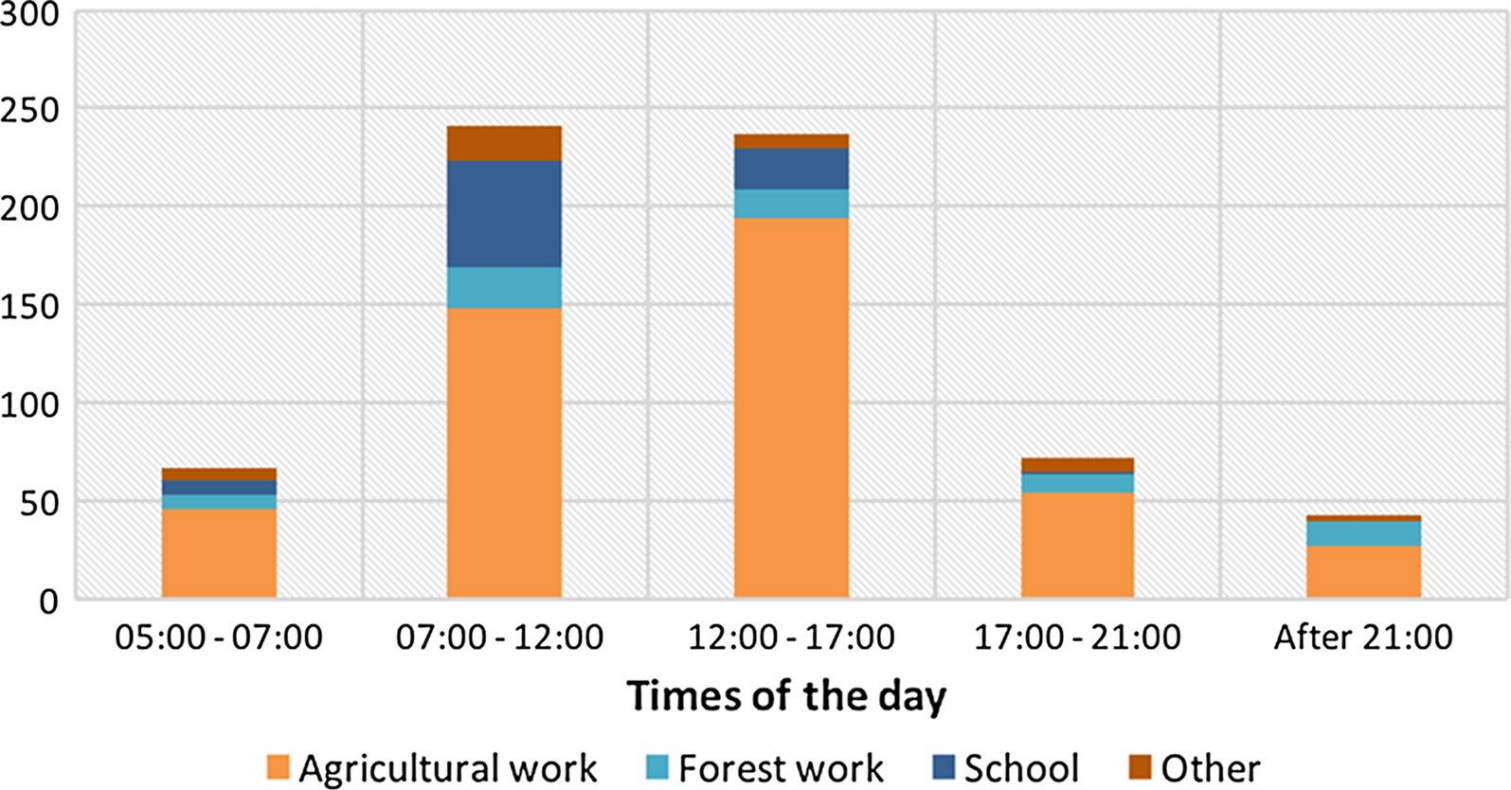
Location of residents in Ou Chra and Pu Cha, when away from their village

During the baseline survey, the temporal and spatial dimensions of routines of all household occupants (n = 448) were mapped to understand potential exposure to mosquito bites and gaps in protection. The settlements tended to be sparsely populated during the day (Fig. 2) as farmers would leave home early in the morning to reach their rice fields or farms, tend cattle or work in the forest, while children went to school (Fig. 3), but after dark most residents were home, and the community population peaked.

In the evening, when malaria vectors may be active, residents spent a considerable time outdoors, resting in hammocks or engaging in social activities. By 21:00, the large majority of villagers were inside their houses (Fig. 2) and 59.8% were in bed (Table 2), where ITNs were reportedly used (Table 3). However, 40.2% stayed up longer (both indoors and outdoors), increasing their risk of exposure to mosquito bites. In addition, of residents who were away in the night preceding the interview, 28.6% (n = 12/42) slept in the deep forest where risk of malaria transmission is greater [34, 35].

**Table 2:** Self-reported usual sleeping time in different age groups

	n	Time to go to sleep n (%)		
		Before 19:00	Between 19:00 and 21:00	Later
Age group (years)				
< 5	49	4 (8.2)	40 (88.9)	5 (11.1)
5–15	123	–	85 (69.1)	38 (30.9)
16–49	146	–	69 (47.3)	77 (52.7)
> 49	27	–	10 (37.0)	17 (63.0)
Total	341	4 (1.2)	204 (59.8)	137 (40.2)

**Table 3:** Reported measures taken against mosquito bites

	Inside the house n (% of cases)	Outside the house n (% of cases)	In the farm or forest n (% of cases)
ITN	83 (96.5)	8 (11.3)	15 (17.2)
Hammock net	–	1 (1.4)	11 (12.6)
Burn incense	2 (2.3)	2 (2.8)	–
Burn mosquito coil	8 (9.3)	2 (2.8)	2 (2.3)
Burn leaves	1 (1.2)	26 (36.6)	27 (31.0)
Indoor sprays	7 (8.1)	–	–
Topical repellent	–	2 (2.8)	–
Wear long clothes	3 (3.5)	24 (33.8)	59 (67.8)
Clear vegetation	5 (5.8)	24 (33.8)	–
Cover water jars	–	2 (2.8)	–
Others	3 (3.5)	5 (7.0)	–
None or don’t know	–	–	–
Total	112 (130)	96 (135)	114 (131)

### Perceptions about mosquito bites and protective methods

**Fig. 4:**
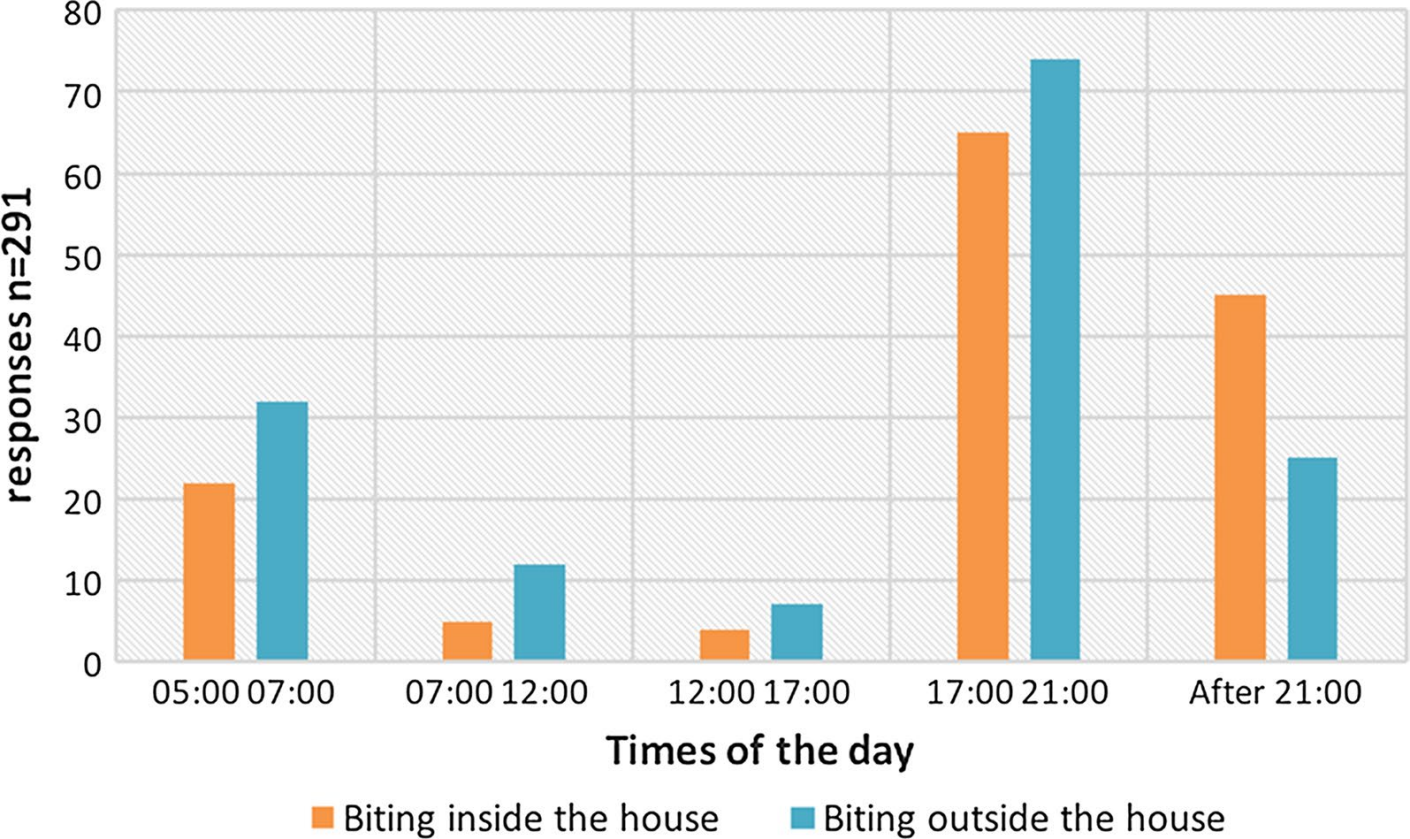
Reported biting times inside and outside the house

Of all respondents at baseline (n = 87), 93.1% (n = 81) reported they had recently been bitten by mosquitoes in the village. The area underneath the elevated house on stilts, where people spent most of the daytime when they were at home, was indicated as the place where outdoor mosquito bites occurred most frequently. Furthermore, 50.6 and 59.5% of respondents noted that bites occurred “very often” inside the house and in the outdoor area surrounding the house, respectively. Indoor biting was reported to occur more frequently in the evening before sleeping time, followed by at night, while outdoor biting occurred more frequently in the early morning (Fig. 4).

Awareness that mosquito bites can spread disease was very high in the study population (98.9%; n = 86/87). When prompted to give further explanations, 85.1% (n = 74) mentioned malaria (*krun chanh*) and associated it with symptoms such as high fever, chill, headache, diarrhea, or headache, while 8% (n = 7) listed correct symptoms without naming a specific disease. Of those who named malaria, 20.3% (n = 15/74) also mentioned that mosquito bites can cause dengue fever (*krun chiam*). Of all cited information sources on malaria (n = 106), the health centre/post (35.8%) was the most frequent, followed by the local VMW (28.3%), non-governmental organizations (13.2%), personal experience (10.4%), radio advertisement (5.7%), private providers (3.8%) and other information sources such as relatives (2.8%). Only a minority of respondents demonstrated incorrect understanding of disease causation, with 6.9% (n = 6/87) stating that mosquitoes can cause both malaria and typhoid fever (*krun pou vien*) and 1.1% (n = 1/87) reporting that malaria can be transmitted by drinking unclean water.

The large majority of respondents reported using protective methods against mosquito bites inside the house (98.9%), outside the house (81.6%) and in the rice field, farm or forest (91.7%). In both villages, bed nets were the most common form of protection inside the house (96.5%), followed by mosquito coils (9.3%), indoor sprays (8.1%), and traditional methods such as burning incense (2.3%) (Table 3). Of all nets across the study households (n = 171), 89.3% were reportedly hung inside the house in the villages (Table 4), while a minority were installed under or near the house (5.7%) or in the rice field or farm (5%). The nets distributed as part of a programme (either through the local VMW, health centre, or non-governmental organization) were allocated free of cost, although a small payment of 1000 riels (US$ 0.25) or less was often given as a voluntary, informal contribution to show appreciation and gratitude. Nets purchased in the private sector (either at the market, local shop or from an itinerant seller) accounted for 13.6% of all nets in the villages, and had a mean cost of 30,360 riels (US$ 7.6), range US$ 3.7–16.2. Finally, a discrepancy between the number of reported and observed nets was found, with only 70.2% of ITNs that were reportedly used in the village being observed.

**Table 4:** Characteristics of all nets in the households

	Total	Ou Chra	Pu Char
	n (%)	n (%)	n (%)
Nets in household	171 (100)	60 (100)	111 (100)
Observed	120 (70.2)	38 (63.3)	82 (73.9)
Not observed	51 (29.8)	22 (36.7)	29 (26.1)
Source of net	169 (100)	58 (100)	111 (100)
Government/NGO	138 (81.7)	46 (79.3)	92 (82.9)
Shop/market	11 (6.5)	5 (8.6)	6 (5.4)
Itinerant seller	12 (7.1)	4 (6.9)	8 (7.2)
Other	3 (1.7)	3 (5.2)	0
Don’t know	5 (3.0)	0	5 (4.5)
Price of net	171 (100)	60 (100)	111 (100)
Not paid	110 (64.3)	25 (41.7)	85 (76.6)
≤ 1000	31 (18.1)	23 (38.3)	8 (7.2)
≥ 15,000 to < 25,000	5 (2.9)	2 (3.3)	3 (2.7)
≥ 25,000 to < 35,000	9 (5.3)	4 (6.7)	5 (4.5)
≥ 35,000	8 (4.7)	2 (3.3)	6 (5.4)
Do not know	8 (4.7)	4 (6.7)	4 (3.6)
Hung where?	159 (100)	50 (100)	109 (100)
Inside the house	142 (89.3)	46 (92.0)	96 (88.1)
Outside the house	9 (5.7)	3 (6)	6 (5.5)
In the field	8 (5)	1 (2)	7 (6.4)

A variety of different methods were used to prevent mosquito bites outdoors (Table 3). In the area surrounding the house, the prevention method most commonly cited were burning leaves (36.6%), clearing vegetation (33.8%) and wearing long clothes (33.8%). In the farm or forest, wearing long clothes was the most common protective method (67.8%), followed by burning leaves (31.0%), using bed nets (17.2%) and hammock nets (12.6%). Of all participants, 80.5% (n = 70/87) said they would like to use additional protective tools. When asked to specify the type of protective tool, mosquito spray was the most frequent answer (32.8%; n = 19/58), followed by coil (20.7%), light traps (15.5%), additional nets (10.3%), topical repellents (6.9%) and spatial (5.2%) repellents. The remaining answers (10.3%) were vague, for example “anything that kills mosquitoes”.

### Reception of spatial repellents, willingness to pay and delivery methods

#### Product reception

Spatial repellents were new to the communities before the start of the trial. Participants reported a sparse use of coils or traditional repellency methods such as burning dried leaves. Yet, no households refused to install the product, no emanators were removed from their locations during the experiment and only one dispenser was reportedly moved from the place of initial instalment.

The follow-up survey revealed mixed findings about perceived efficacy and acceptability of the product (Table 5). Overall, the majority of respondents understood the properties of the repellent, with 95.4% of participants (n = 83/87) reporting it was aimed to keep mosquitoes away or prevent mosquito bites. In both villages, most people perceived the emanator as useful, with 47.1% of respondents reporting they had “much fewer” mosquitoes after the first instalment of the product, as opposed to “moderately fewer” mosquitoes or “not at all”. The large majority of respondents (96.5%) reported they perceived no side effects or discomfort from the product or did not know (3.5%).

**Table 5:** Perceptions and preferences about spatial repellents after use: effectiveness, rating, and delivery methods

Questions	Ou Chra n (%)	Pu Cha n (%)	Total n (%)	p value*
Have you had fewer mosquitoes after the instalment of the repellent?				0.001
Yes—much fewer	3 (10)	38 (66.7)	41 (47.1)	
Yes—moderately fewer	15 (50.0)	18 (31.6)	33 (37.9)	
Not at all	8 (26.7)	1 (1.7)	9 (10.3)	
Do not know/other	4 (13.3)	0 (0.0)	4 (4.6)	
How would you rate this product overall?				
Very useful	8 (27.6)	22 (38.6)	30 (34.9)	
Useful	7 (24.1)	29 (50.9)	36 (41.9)	
Not very useful	8 (27.6)	5 (8.8)	13 (15.1)	
Useless	2 (6.9)	0 (0.0)	2 (2.3)	
Do not know	4 (13.8)	1 (1.8)	5 (5.8)	
Are you willing to use the repellent again?				0.423
Yes	28 (93.3)	56 (98.3)	84 (96.6)	
No	1 (3.3)	0 (0.0)	1 (1.2)	
Do not know	1 (3.3)	1 (1.8)	2 (2.3)	
Would you prefer spatial repellents over bed nets				0.104
Yes	8 (26.7)	22 (38.6)	30 (34.5)	
No	20 (66.7)	35 (61.4)	55 (63.2)	
Do not know	2 (6.7)	0 (0.0)	2 (2.3)	
What is your preferred purchase channel?				< 0.001
Health centre/post	16 (55.2)	1 (1.8)	17 (20.0)	
Private provider	0 (0.0)	2 (3.6)	2 (2.4)	
Market	0 (0.0)	1 (1.8)	1 (1.2)	
VMW	8 (27.6)	34 (60.7)	42 (49.4)	
Village chief	5 (17.2)	15 (26.8)	20 (23.5)	
Do not know	0 (0.0)	3 (5.4)	3 (3.5)	

* Fisher’s exact test

Most respondents in both villages said they would be willing to use the emanator again (96.6%) and 69.8% would recommend other villagers to use it, but 63.2% (n = 55/87) said they would not prefer spatial repellents over bed nets. When asked to provide further explanations, 65.5% of respondents (n = 36/55) said that nets are safer or more reliable than the spatial repellents; others noted they were accustomed to bed nets and, therefore, unwilling to change their habits. Preferences for other products, such as sprays or coils, were less clear. Those who had a preference for repellents and provided further explanations said the repellent was “easy” to apply (30.8%; n = 8/26), did not produce smoke or “bad smell” like sprays (30.8%), and lasted longer (15.4%). Overall, the repellent had a more positive reception in Pu Cha (Table 5), but we could not explain this finding on the basis of collected data.

#### Willingness to pay

Spatial repellents were provided free of charge to all accessible families in the villages, with a mean distribution of 2.5 units per household (range 1–6). Since this product was not available in the market or through the health system, WTP per unit, conventionally defined as the “maximum sum an individual (or a government) is willing to pay to acquire some good or service, or the maximum sum an individual (or government) is willing to pay to avoid a prospective loss” [36] was explored. In both study locations, the majority of respondents were willing to purchase the repellent for an average price of 1268.99 Cambodian riels (US$ 0.32) per unit. Socioeconomic status did not have a statistically significant effect on mean scores of willingness to pay (Table 6). An independent t test found no significant association between gender and WTP, t (77) = − 1.0248, p = 0.309.

**Table 6:** Mean willingness to pay by socio-economic status, Cambodian Riel (US Dollar)

	Mean	St. dev.	Median	Range	p value
Willingness to pay					0.1413*
1 (poorest)	1219.23 (0.30)	1340.16 (0.33)	750 (0.19)	200 (0.05)–5000 (1.25)	
2 (poor)	1338.46 (0.33)	741.93 (0.19)	1000 (0.25)	300 (0.07)–3000 (0.75)	
3 (least poor)	1250.00 (0.31)	1002.40 (0.25)	1000 (0.25)	250 (0.06)–5000 (0.75)	
Total	1268.99 (0.32)	1000 (0.26)	1000 (0.25)	200 (0.05)–5000 (1.25)	

* Kruskal–Wallis test (Χ^**2**^ = 3.914)

#### Procurement

Most respondents indicated they would prefer to purchase the repellent from VMWs, followed by the village chief’s house and the local health centre (Table 5). Only one respondent said he would like to find it at the market. Thus, a clear preference for local procurement emerged. Respondents who preferred local procurement further explained their choice by stressing they would like to find the product “near home” (60.2%) and save the money on gasoline required to travel to shops and the market. In both research sites, 45.3% of those who preferred VMWs (n = 19/42) noted that VMWs were already familiar with malaria and thus could advise on product utilization. However, a statistically significant difference between preferences in the two villages was observed, particularly with regard to the choice of the local health centre/post as a procurement method. This issue was not investigated further, but it is reasonable to assume that the shorter distance between Ou Chra and the local health post may be an explanatory factor.

## Discussion

Over the past 10 years there has been a marked decrease in clinical cases of malaria in Cambodia, which has coincided with large-scale distribution of ITNs and deployment of VMWs in high-risk areas. However, malaria transmission persists in endemic pockets, including areas in which distribution of bed nets has been implemented. The present study aimed to better understand the nature of gaps in protection from potentially infective mosquito bites in one of these contexts, and consequently assess the adequacy, feasibility, and acceptability of a novel tool to address them.

As noted, nearly all respondents reported frequent mosquito bites both inside and outside the house, with a peak in the evening before sleeping times when most people were in the village. The parasitological survey that was conducted in conjunction with this study found relatively low rates of malaria transmission in both villages; however, the entomological survey identified fourteen anopheline species from light-trap collections in Ou Chra and nine from Pu Cha [25], including *Anopheles dirus* and *Anopheles minimus*, the known primary malaria vectors in Cambodia [37]. *Anopheles dirus* was the most common anopheline caught in light-traps in Ou Chra and the second most common species in Pu Cha, where *Anopheles maculatus* was the most common species. While the entomological survey did not collect data on biting times and feeding behaviours, past studies in forested villages in Cambodia and other parts of Southeast Asia found that these vectors can bite in the early evening [18, 38]. Further, females of *An. dirus* are primarily exophilic and exophagic but may enter houses to feed, especially open houses built directly on the ground such as those found in Ou Chra and Pu Cha [39]. Thus, it is clear that indoor and outdoor risk of residual malaria transmission exists in the villages, particularly in the households closer to the edge of the forest, where higher densities of *An. dirus* were found in the entomological survey.

Spatial repellents could be an effective approach to enhance environmental protection in such contexts, if units are installed properly so as to create a “safe zone” in the volume of space occupied by human hosts. The utilization of metofluthrin based repellents may also help reduce the transmission of dengue fever, which is endemic throughout Cambodia [13]. Novel repellents, such as metofluthrin, also have the potential to promote equity in access to health resources, as the active ingredient can be applied on paper or resin emanators [40]. In the two villages, electric power was available in 57.5% of households, thus vapour-phase repellents would be more equitable than other types of spatial repellents that require an electric source, such as mosquito mats or light traps. However, some reservations should be noted. While spatial repellents have the potential to enhance significantly the effect of ITNs where no treatable surfaces exist, combined indoor use of both measures is not recommended. As Killeen and Moore pointed out, “if repellents are also used indoors in settings where LLINs are common, mosquitoes can be deterred from exposure to fatal contact with products so that overall protection is attenuated” [7]. As a result, spatial repellents would not be suitable to fill indoor protective gaps when nets are not used, unless a full net replacement plan is implemented. Findings from this study suggest this would be a challenging policy shift, given participants’ satisfaction and familiarity with bed nets, which were perceived to provide a more immediate and tangible form of protection.

Programmatic use of spatial repellents in peridomestic areas outside the house could be a more promising policy option, especially given the importance of outdoor malaria transmission in Cambodia [4, 18]. Other methods have been suggested to target outdoor biting in Cambodia, including insecticide-treated hammock nets [41] and topical repellents [42]. In comparison with these approaches, spatial repellents require a modicum of compliance, while protecting multiple subjects within their range of action. Further, when applied on a wide scale, it is possible that an area-wide effect could be obtained, such as is observed with mosquito nets or topical repellents [43, 44]. Spatial repellents are also highly portable and therefore could be moved into different settings, including the farm or the forest, where the risk of malaria transmission is higher. However, evidence about the efficacy of vapour-phase repellents against malaria vectors is still limited and inconclusive [7]. A recent study in Pailin province, in western Cambodia, found that metofluthrin emanators significantly reduced outdoor landing rates of *An. minimus* and *An. maculatus* [45], but the effects of the repellent on *An. dirus* were unclear. Findings from the surveys conducted in combination with this study were also inconclusive about the entomological and parasitological efficacy of the repellent in the study locations [25].

The acceptability and perceived value of the emanator was another key question in our investigation. During the follow-up survey, the study population liked the product, most respondents demonstrated a good understanding of its properties and use, and said they would like to have additional protective tools against mosquito bites. However, a high level of acceptability and understanding does not necessarily translate into uptake and sustained use of a new preventive method. For example, past evaluations found that only 40–60% of participants in western Cambodia reported sleeping under insecticide-treated nets, despite large-scale distribution and intense communication efforts [46]. Similarly, a recent survey in Kampong Cham province, in central Cambodia, found a significant gap between high levels of awareness regarding the transmission of dengue and vector control practices in the same locations, suggesting that an education campaign alone is unlikely to promote behavioural change unless it is incorporated in a more comprehensive participatory approach (Kumaran et al. pers. comm.).

Cost might be another important barrier to uptake and regular utilization of the product. While socioeconomic status was not significantly associated with increased WTP, most respondents were prepared to pay only a fraction of the consumer retail price of a similar product available in the market (typically around US$ 5 per unit, but this can vary considerably by country). Therefore, widespread use in Cambodia would require the development of a dedicated low-cost product whose price can be afforded and sustained in poor settings. In addition, some form of subsidization and negotiations with the manufacturers might be needed. Past experiences in Cambodia can offer useful lessons; in 2004, for example, access to subsidized ACT, Malarine^®^, was scaled up at private retail outlets after a study found that the majority of recipients were not willing to pay the initial price of 7900 riel (US$ 1.93) for the adult package and 4900 riel (US$ 1.20) for the child package [47]. Social marketing activities were implemented to promote consumer awareness, and while uptake of products was initially slow, there has been a marked and progressive increase in market sales ever since and improvements in health-seeking behaviour [48]. A similar approach could support the introduction of spatial repellents in Cambodia, including promotional activities and packaging strategies to attract consumers and increase awareness. Yet, donor funding to support subsidization may be challenging to achieve as long as spatial repellents do not have a formal WHO recommendation, backed by conclusive evidence of their epidemiological efficacy, especially in outdoor environments.

Lastly, findings from this study suggest some reflections on delivery methods. In the follow-up survey, participants reported a clear preference for local availability. This is not surprising given that the product would require monthly replacement, and travel to the market town is costly and difficult, especially during the rainy season. Distribution of repellents from local VMWs, as indicated by many participants, would be an appropriate delivery channel and would enable alignment with the priorities of the national malaria programme and meaningful operationalization, especially as VMWs are already established in remote endemic areas, where access to towns and health facilities is most difficult. In addition, past experiences suggest that the allocation of new responsibilities, such as the involvement in participatory activities to promote product utilisation, is likely to increase VMWs motivation [49] and dependability in user communities.

### Study limitations

The small size of the study population is perhaps the most important limitation of this study. In addition, differing practices and beliefs could potentially be observed in other endemic areas and ethnic minorities. Long-term ethnographic work in the villages and the use of qualitative methods such as focus group discussions were also not possible due to time and resource constraints. This would have been a valuable addition to the research design, enabling deeper understanding of protective gaps in the study locations and further insights on the feasibility and acceptability of the product. Finally, the product was not tested in environments outside the villages where high risk of malaria transmission exists, such as the deep forest or the shelters where some farmers stay overnight in the harvesting season.

## Conclusion

Widespread use of spatial repellents in the study locations would not fill all protective gaps, but their application in outdoor environments has the potential to enhance current vector control strategies. Further research is needed to inform policy development and programme implementation, including more comprehensive and conclusive evidence on the efficacy of novel spatial repellents in different environmental conditions. It is hoped this study will generate further interest in the evaluation and uptake of spatial repellents for malaria vector control, while contributing to recent efforts towards a better understanding of the social context of residual malaria transmission in Cambodia and the role of human mobility [18].

## Additional files

**Additional file 1.** Questionnaire for the baseline survey.

**Additional file 2.** Data collection tool for spatial mobility patterns.

**Additional file 3.** Questionnaire for the follow-up survey.

## Abbreviations

ACT: artemisinin-based combination therapy
IRS: indoor residual spraying
ITNs: insecticide-treated bed nets
LLINs: long-lasting insecticide-treated bed nets
PCA: principal component analysis
RDT: rapid diagnostic test
VMWs: village malaria workers
WTP: willingness to pay
