# Supplementary material for: Field assessment of a novel spatial repellent for malaria control: a feasibility and acceptability study in Mondulkiri, Cambodia
Source: Malar J. 2017 Oct 13;16:412. doi: 10.1186/s12936-017-2059-6 (PMC5640900; doi:10.1186/s12936-017-2059-6)
Supplement: Supplementary file 1 — Additional file 1. Questionnaire for the baseline survey. [file 12936_2017_2059_MOESM1_ESM.docx]

**FRAME Project: Mosquito-bites, disease awareness, and prevention**

**Research site (village and household ID):**

**Name of respondent:**

**Age and gender:**

**Field researchers:**

**Date and time:**

| **Section A. OPENING** | | |
| --- | --- | --- |
| *Could you please tell me a little about yourself...* | | |
|  | How long have you lived in this village for? |  |
| **A2** | What is your main occupation at the moment? |  |
| **Section B. MOSQUITO BITES** | | |
| *Thank you. I would like to ask you some questions about mosquito bites in your house...* | | |
|  | Have you been bitten by mosquitoes inside your house recently? | 1. Yes 2. No **(Skip to B4)** 3. Don’t know **(Skip to B4)** |
|  | How often?  **Prompt. Circle one answer.** | 1. Very often 2. Sometimes 3. Rarely |
|  | At what time of the day?  **Do not read out.**  **Multiple responses allowed.** | 1. Early morning  2. Morning  3. Afternoon  4. Late afternoon  5. Evening  6. At night  7. Don’t know |
|  | Have you been bitten by mosquitoes while you are outside around your house recently? | 1. Yes 2. No **(Skip to C1)** 3. Don’t know **(Skip to C1)** |
|  | How often?  **Prompt. Circle one answer.** | 1. Very often 2. Sometimes 3. Rarely |
|  | Where about? |  |
|  | At what time of the day?  **Do not read out.**  **Multiple responses allowed.** | 1. Early morning  2. Morning  3. Afternoon  4. Late afternoon  5. Evening  6. At night  7. Don’t know |
| **Section C. DISEASE AWARENESS** | | |
| *Thank you. I would like to ask you further questions about mosquitoes...* | | |
|  | Do you think mosquito bites can make people sick? | 1. Yes 2. No **(Skip to D1)** 3. Don’t know **(Skip to D1)** |
|  | How sick? What kind of sickness/disease?  **PROBE: severity; duration; symptoms** |  |
|  | How do you know that? |  |
| **Section D. BITING PREVENTION METHODS** | | |
| *Thank you. We would like to ask you some questions about mosquito bites and prevention...* | | |
|  | Do you do anything to prevent mosquito bites inside your house? | 1. Yes  2. No **(If SO)**: Why not?  ___________________________________________  ­­­­­­­­­__________________________________ **(Skip to D5)**  3. Don’t know **(Skip to D5)** |
|  | What do you do?  **Do not read out.**  **Multiple responses allowed.** | 1. Boil water 2. Mosquito net 3. Burn incense 4. Burn coil 5. Skin repellent 6. Spray house with insecticide 7. Other (specify)___________ 8. Don’t know |
|  | Do you do anything to prevent mosquito bites outside around the house? | 1. Yes  2. No **(If SO)**: Why not?  ___________________________________________  ­­­­­­­­­__________________________________ **(Skip to D9)**  3. Don’t know **(Skip to D9)** |
|  | What do you do?  **Do not read out.**  **Multiple responses allowed.** | 1. Mosquito net 2. Burn leaves 3. Burn incense 4. Burn coil 5. Skin repellent 6. Clear vegetation around the house 7. Cover water jars 8. Other (specify)_____________________ 9. Don’t know |
|  | If you go to the forest/rice fields/chamkar, do you do anything to prevent mosquito bites? | 1. Yes  2. No **(If SO)**: Why not?  ___________________________________________  ­­­­­­­­­__________________________________ **(Skip to D13)**  3. Don’t know **(Skip to D13)** |
|  | What do you do?  **Do not read out.**  **Multiple responses allowed.** | 1. Bed-net 2. Hammock net 3. Burn leaves 4. Burn incense 5. Burn coil 6. Skin repellent 7. Other (specify)____________________ 8. Don’t know |
|  | Would you like to use any other methods or products to prevent mosquito bites? | 1. Yes 2. No **(Skip to E1)** 3. Don’t know **(Skip to E1)** |
|  | What methods or product? | 1. (Specify)_________ 2. Don’t know |

| **Section E. BED-NETS RECORD** | | | | |
| --- | --- | --- | --- | --- |
| *I would like to ask you about each bed-net that you have in the household.*  *This includes all nets that were owned and present in the household last night.* | | | | |
| **First ask questions E1 TO E7 for net 1, then for net 2, etc...**  **If there are more than three bed-nets in the household, use additional sheets.** | | | | |
|  | **How many nets does your household have?** | [ ] [ ] | | |
|  |  | **Net 1** | **Net 2** | **Net 3** |
|  | **INTERVIEWER ONLY: Ask if you can see the nets in the household. Did you observe the net?** | 1. Observed  2. Not observed | 1. Observed  2. Not observed | 1. Observed  2. Not observed |
|  | Where did your household obtain this net? | 1. Family/friend  2. Government  3. NGO  4. Shop/Market  5. Itinerant seller  6. Other:  ________________  7. Don't know | 1. Family/friend  2. Government  3. NGO  4. Shop/Market  5. Itinerant seller  6. Other:  ________________  7. Don't know | 1. Family/friend  2. Government  3. NGO  4. Shop/Market  5. Itinerant seller  6. Other:  ________________  7. Don't know |
|  | Did you have to pay for this net? | 1. Riel____________  2. No  3. Don’t know | 1. Riel____________  2. No  3. Don’t know | 1. Riel____________  2. No  3. Don’t know |
|  | Did anyone sleep under this net last night? | 1. Yes  2. No **(Skip to F1)**  3. Not sure **(Skip to F1)** | 1. Yes  2. No **(Skip to F1)**  3. Not sure **(Skip to F1)** | 1. Yes  2. No **(Skip to F1)**  3. Not sure **(Skip to F1)** |
|  | Who slept under this net last night? | Name(s): | Name(s): | Name(s): |
|  | Where was this net hung last night? |  |  |  |

| **Section F. CLOSING** | | |
| --- | --- | --- |
| *Thank you so much…* | | |
| **F1** | Is there anything we haven’t discussed that you would like to say? |  |

Thank you very much. Can we ask you some additional information about yourself and your household?

CONDUCT HOUSEHOLD QUESTIONNAIRE
