# Supplementary material for: Field assessment of a novel spatial repellent for malaria control: a feasibility and acceptability study in Mondulkiri, Cambodia
Source: Malar J. 2017 Oct 13;16:412. doi: 10.1186/s12936-017-2059-6 (PMC5640900; doi:10.1186/s12936-017-2059-6)
Supplement: Supplementary file 2 — Additional file 2. Data collection tool for spatial mobility patterns. [file 12936_2017_2059_MOESM2_ESM.doc]

| **Section A: Household roster**  *Explain you are going to ask some questions about the household where they are living now - Including temporary visitors that were in the household last night.* |
| --- |

| No. | Name | Sex | Age  *(If under 1 year, write “00”)* | Relation-ship to head of household  **INSERT code*** | Residency  status | Highest grade of education attained?  **INSERT code**** | Occupation  (*If has more than one occupation then write the code for* ***main*** *occupation)*  **INSERT code***** |  | | | | |
| --- | --- | --- | --- | --- | --- | --- | --- | --- | --- | --- | --- | --- |
|  |  |  |  |  |  |  |  | Early morning | Morning | Afternoon | Evening | Night |
| 01 |  | M F | |__|__| | |___|___| | 1. Resident  2. Visitor | |___| | |___|  _____________ | |___| ________ | |___| ________ | |___| ________ | |___| ________ | |___| ________ |
| 02 |  | M F | |__|__| | |___|___| | 1. Resident  2. Visitor | |___| | |___|  _____________ | |___| ________ | |___| ________ | |___| ________ | |___| ________ | |___| ________ |
| 03 |  | M F | |__|__| | |___|___| | 1. Resident  2. Visitor | |___| | |___|  ____________ | |___| ________ | |___| ________ | |___| ________ | |___| ________ | |___| ________ |
| 04 |  | M F | |__|__| | |___|___| | 1. Resident  2. Visitor | |___| | |___|  ____________ | |___| ________ | |___| ________ | |___| ________ | |___| ________ | |___| ________ |
| 05 |  | M F | |__|__| | |___|___| | 1. Resident  2. Visitor | |___| | |___|  ____________ | |___| ________ | |___| ________ | |___| ________ | |___| ________ | |___| ________ |
| 06 |  | M F | |__|__| | |___|___| | 1. Resident  2. Visitor | |___| | |___|  ____________ | |___| ________ | |___| ________ | |___| ________ | |___| ________ | |___| ________ |
| 07 |  | M F | |__|__| | |___|___| | 1. Resident  2. Visitor | |___| | |___|  ____________ | |___| ________ | |___| ________ | |___| ________ | |___| ________ | |___| ________ |
| 08 |  | M F | |__|__| | |___|___| | 1. Resident  2. Visitor | |___| | |___|  _____________ | |___| ________ | |___| ________ | |___| ________ | |___| ________ | |___| ________ |
| 09 |  | M F | |__|__| | |___|___| | 1. Resident  2. Visitor | |___| | |___|  _____________ | |___| ________ | |___| ________ | |___| ________ | |___| ________ | |___| ________ |
| 10 |  | M F | |__|__| | |___|___| | 1. Resident  2. Visitor | |___| | |___|  ____________( | |___| | |___| | |___| | |___| | |___| |

| ***Code for relationship to household head** | ****Code for highest level of education attained** | *****Code for occupation** | ******Code for the use of household spaces** |
| --- | --- | --- | --- |
| 01 = Head of household  02 = Spouse  03 = Son or daughter  04 = Son or daughter-in-law  05 = Grandchild  06 = Parent  07 = Parent in-law  08 = Brother or sister  09 = Other relative  10 = Not related | 0 = Never attended school  1= Some primary  2=Completed primary  3=Some secondary  4=Completed secondary  5=More than secondary  6=Don’t know | 1 = Farmer own land  2 = Hired plantation or farm labourer  3 = Military or police  4 = Own business (eg shop owner but not pharmacy/clinic)  5 = Health worker  6 = Look after home  7 = Child or student  8 = Retired or disabled  9 = Other **(specify)** | 01 = S/he stayed mainly inside the house  02 = S/he stayed mainly outside around the house  03 = S/he was away **(specify)**  04 = I don’t know/not sure |
