# Supplementary material for: Field assessment of a novel spatial repellent for malaria control: a feasibility and acceptability study in Mondulkiri, Cambodia
Source: Malar J. 2017 Oct 13;16:412. doi: 10.1186/s12936-017-2059-6 (PMC5640900; doi:10.1186/s12936-017-2059-6)
Supplement: Supplementary file 3 — Additional file 3. Questionnaire for the follow-up survey. [file 12936_2017_2059_MOESM3_ESM.docx]

**FRAME Project Cambodia: Follow up Questionnaire**

| **Section A. Use and reception of the dispenser.** | | | |
| --- | --- | --- | --- |
|  | Do you know what the specific use of this product is?  **[SHOW THE DISPENSER]**  **Do not read out. Multiple answers allowed.** | 1. To keep mosquitoes away from the house  2. To kill mosquitoes  3. To prevent infectious diseases  4. Other: **Specify** ______  5. Don’t know  **[If respondent doesn’t know the answer or is completely out of track ask respondent if they know someone else in the household who may be better informed. If this is not possible, explain the use of the emanator and continue the interview].** | |
|  | Did you or someone else in your household remove any dispensers during the study period? | 1. Yes: **PROBE:** Can you tell me why?  __________________________________________  __________________________________________  2. No  3. Don’t know | |
|  | Did you or someone else in your household move any dispensers from the place they were initially applied to another place in your house? | 1. Yes: **PROBE:** Can you tell me why?  __________________________________________  __________________________________________  2. No  3. Don’t know | |
|  | Do you know how long the dispenser is effective for? | 1. Yes: **Specify** ______  2. No  3. Don’t know | |
|  | Have you had fewer mosquitoes inside your house after the dispenser was applied?  **Prompt. Circle one answer.** | 1. Yes, much fewer  2. Yes, moderately  3. Not at all  4. Don’t know  5. Other (Specify______________) | |
|  | Have you had fewer mosquito bites inside your house after the dispenser was applied?  **Prompt. Circle one answer.** | 1. Yes, much fewer  2. Yes, moderately  3. Not at all  4. Don’t know  5. Other (Specify______________) | |
|  | Have you had any side effects or discomfort with the dispenser?  **Do not read out. Multiple answers allowed.** | 1. Foul smell  2. Sneezing  3. Not at all  4. Don’t know  5. Other __________________ | |
|  | Would you prefer using this product over **bed-nets** for mosquito-bites prevention? | 1. Yes  2. No  3. Don’t know | **[IF YES or NO]:** Why?  _______________________________  _______________________________  _______________________________ |
|  | Would you prefer using this product over **sprays** for mosquito-bites prevention? | 1. Yes  2. No  3. Don’t know | **[IF YES or NO]:** Why?  _______________________________  _______________________________  ______________________________ |
|  | Would you prefer using this product over **coils** or **incense** for mosquito-bites prevention? | 1. Yes  2. No  3. Don’t know | **[IF YES or NO]:** Why?  _______________________________  _______________________________  _______________________________ |
|  | How would you rate this product overall?  **Prompt. Circle one answer.** | 1. Very useful  2. Useful  3. Not very useful  4. Useless  5. Don’t know | |

| **Section B. Willingness to pay.** | | | |
| --- | --- | --- | --- |
|  | Would you continue to use this product? | 1. Yes  2. No **→ END OF INTERVIEW**  3. Don’t know | |
|  | Would you be willing to pay 1,000 Riels for the dispenser, bearing in mind that each dispenser must be replaced with a new one every month? | 1. Yes  2. No **→ GO TO QUESTION B4**  3. Don’t know | |
|  | Would you be willing to pay 2,000 Riels for each dispenser? | 1. Yes **→ GO TO QUESTION B5**  2. No **→ GO TO QUESTION B5**  3. Don’t know **→ GO TO QUESTION B5** | |
|  | Would you be willing to pay 500 Riels for each dispenser? | 1. Yes  2. No  3. Don’t know | |
|  | What is the highest price you would be willing to pay for one dispenser? | 1. Riel ___________  2. Don’t know | |
|  | Would you recommend others to use this product? | 1. Yes  2. No  3. Don’t know | **IF YES:** Who would you recommend this product?  ___________________________  ___________________________  ___________________________ |

| **Section C. Delivery mechanisms.** | | |
| --- | --- | --- |
|  | Where would you like to find this product available as first choice?  **Prompt. Circle one answer.** | 1. Market  2. Health centre  3. Health post  4. Private provider in village  5. Village Health Volunteers/Village Malaria Workers  6. Village chief house  7. Don’t know |
|  | Why? |  |

**Thank you very much for your time and help.**

**END OF INTERVIEW**
